# Assessment of the structural and functional impact of in-frame mutations of the *DMD* gene, using the tools included in the eDystrophin online database

**DOI:** 10.1186/1750-1172-7-45

**Published:** 2012-07-09

**Authors:** Aurélie Nicolas, Céline Lucchetti-Miganeh, Rabah Ben Yaou, Jean-Claude Kaplan, Jamel Chelly, France Leturcq, Frédérique Barloy-Hubler, Elisabeth Le Rumeur

**Affiliations:** 1 Université de Rennes 1, Rennes, France; 2 UMR CNRS 6026, Equipe RMN-ILP, Faculté de Médecine, CS 34317, Rennes Cedex, 35043, France; 3 Université Européenne de Bretagn, 5, Boulevard Laënnec, Rennes, 35000, France; 4 UMR CNRS 6026, Equipe SP@RTE, Campus Beaulieu, Rennes cedex, 35042, France; 5 Département de recherche clinique, Institut de Myologie, GH Pitié-Salpétrière, Paris,, France; 6 Laboratoire de Biochimie et Génétique Moléculaire–Hôpital Cochin,, Paris, France; 7 Institut Cochin, CNRS UMR 8104, Inserm U 1016, Université Paris Descartes, Paris,, France; 8 Faculté de Médecine Paris Descartes, CHU Cochin, Paris, France; 9 IFR 140 Biosit, Plate-forme Amadeus, Université de Rennes1, Rennes, France; 10 UMR CNRS 6290, Institut de Génétique et Développement, CS 34317, Rennes cedex, 35043, France

**Keywords:** Dystrophin, *DMD* gene mutations, Spectrin-like repeats, Duchenne muscular dystrophy, Becker muscular dystrophy, Phenotype-genotype correlation

## Abstract

**Background:**

Dystrophin is a large essential protein of skeletal and heart muscle. It is a filamentous scaffolding protein with numerous binding domains. Mutations in the *DMD* gene, which encodes dystrophin, mostly result in the deletion of one or several exons and cause Duchenne (DMD) and Becker (BMD) muscular dystrophies. The most common *DMD* mutations are frameshift mutations resulting in an absence of dystrophin from tissues. In-frame *DMD* mutations are less frequent and result in a protein with partial wild-type dystrophin function. The aim of this study was to highlight structural and functional modifications of dystrophin caused by in-frame mutations.

**Methods and results:**

We developed a dedicated database for dystrophin, the eDystrophin database. It contains 209 different non frame-shifting mutations found in 945 patients from a French cohort and previous studies. Bioinformatics tools provide models of the three-dimensional structure of the protein at deletion sites, making it possible to determine whether the mutated protein retains the typical filamentous structure of dystrophin. An analysis of the structure of mutated dystrophin molecules showed that hybrid repeats were reconstituted at the deletion site in some cases. These hybrid repeats harbored the typical triple coiled-coil structure of native repeats, which may be correlated with better function in muscle cells.

**Conclusion:**

This new database focuses on the dystrophin protein and its modification due to in-frame deletions in BMD patients. The observation of hybrid repeat reconstitution in some cases provides insight into phenotype-genotype correlations in dystrophin diseases and possible strategies for gene therapy. The eDystrophin database is freely available: http://edystrophin.genouest.org/.

## Background

The *Duchenne Muscular Dystrophy (DMD)* gene, located on the short arm of the X chromosome (at Xp21.2), is the largest known gene in humans. It has an open reading frame of ~11.055 kb, containing 79 exons (Mendelian Inheritance in Man [MIM: 300377]) [1], and transcription from seven tissue-specific promoters leads to the synthesis of 16 isoforms of the dystrophin protein. In humans, dystrophin diseases are caused by mutations in the *DMD* gene and include the allelic phenotypes of Duchenne muscular dystrophy (DMD) [OMIM:310200], Becker muscular dystrophy (BMD) [OMIM:300376] and X-linked dilative cardiomyopathy (XLDCM) [OMIM:302045] [1-3].

Dystrophin is present at the internal face of the plasma membrane in many tissues, including skeletal, cardiac and smooth muscle, and in various central nervous system cells. Dystrophin is highly conserved in vertebrates, including mouse, chicken and dog, and in invertebrates, such as *Drosophila*[4], and *Caenorhabditis elegans*[5].

The full-length 3685-residue isoform of dystrophin, dp427m, has a molecular weight of 427kDa and is expressed in skeletal and cardiac muscle, where it plays a key role during muscle contraction-relaxation cycles. Dystrophin has four main regions: (i) the N-terminal actin-binding domain (ABD) comprises the first 246 residues; (ii) the central rod domain spans residues 247 to 3045 (accounting for about 76% of the molecule [6,7]), is formed by 24 spectrin-like repeats and four hinges and binds to various partners (filamentous actin, membrane lipids and nitric oxide synthase); (iii) the cysteine-rich domain, from residues 3080 to 3360, binds to the intrinsic membrane protein β-dystroglycan and (iv) the carboxy-terminal domain, comprising the last 325 residues, binds to dystrobrevin and syntrophins (for reviews, see [8,9]). Dystrophin is associated with a large number of proteins, to which it either binds directly or with which it interacts indirectly through intracellular or extracellular proteins. The binding of dystrophin to β–dystroglycan brings it into contact with membrane and extracellular proteins to form the dystrophin-glycoprotein complex (DGC) [10,11]. Dystrophin therefore forms a link between the extracellular matrix and cytoskeletal actin. The function of dystrophin is not completely understood, but its main role is to protect the sarcolemma from rupture during the stresses of muscle contraction [12,13].

Patients with muscular dystrophy have little or no dystrophin, and it has been suggested that this results in the disruption of muscle membranes, which alters calcium-channel activity, thereby strongly increasing intracellular calcium concentration. This ultimately leads to muscle-cell necrosis [13,14], followed by regeneration. The continual cycles of regeneration and necrosis lead to the skeletal muscles being gradually replaced by adipose tissue and unable to sustain any mechanical activity.

The incidence of Duchenne muscular dystrophy (DMD) is about 1 in 3,500 male births. Affected patients have a massively reduced life expectancy and a poor functional prognosis. In most cases, DMD is due to frame-shift mutations in the *DMD* gene, leading to a complete absence or low levels of dystrophin protein (no more than 3% normal levels). This accounts for the severity of the phenotype in all patients, although some variation of disease expression is observed between patients, in terms of motor, respiratory, cardiac and mental functions [15,16]. The expression of the various dystrophin isoforms may depend on the mutation site, potentially accounting, at least in part, for the correlation with the motor and mental status of patients [15]. Becker muscular dystrophy (BMD) is less frequent than DMD, and is usually milder, with slower disease progression. BMD is caused by in-frame deletions or duplications of one or several exons or by splice-site and missense mutations. These mutations lead to the production of various amounts of internally truncated, lengthened, or slightly modified dystrophin molecules. This results in a broad spectrum of clinical severity, ranging from a complete absence of symptoms, through mild disease, to severe clinical conditions similar to DMD [15-25]. According to the reading-frame rule, frame-shifting mutations lead to the severe DMD phenotype, whereas in-frame mutations lead to the less severe BMD phenotype [16,26]. However, there are exceptions to this rule, with certain in-frame mutations resulting in the severe DMD phenotype [27-31]. These mutations are frequently located at the 5’ end of the gene encoding the N-terminus of dystrophin including ABD1, or at the 3’ end of the gene encoding the C-terminal domain, usually in the Cys-rich domain, thereby disrupting the DGC.

The structure and function of dystrophin are poorly resolved at the biological and physiological levels, and it is therefore difficult to establish a detailed phenotype-genotype correlation in BMD patients. Phenotypic differences between patients are thought to depend on the site of the deletion or duplication and the conservation of the reading frame, and such differences have recently been shown to be correlated with the residual amount of dystrophin [32]. Such knowledge is essential to anticipate the effects of current exon-skipping treatments on phenotype restoration in treated DMD patients [33-36].

Two databases of *DMD* human mutations are already freely available online: the Leiden Muscular Dystrophy database [37,38] and the UMD-DMD French database [39,40]. The Leiden Muscular Dystrophy database lists the *DMD* mutations in patients reported in publications or submitted by contributors from around the world and includes some biochemical and phenotypic details. The UMD-DMD database provides molecular and clinical data for patients from France carrying a mutation of the *DMD* gene. Both databases include in-frame and frame-shifting mutations and focus on gene-level information. However, as dystrophin acts at the protein level, a more detailed and comprehensive characterization of the protein produced from genes with in-frame gene mutations is required. Such a characterization is particularly important for comparisons of the structural features and molecular interactions of the mutated protein with those of the wild-type protein. For example, the total absence of dystrophin, or its presence in very small amounts in DMD patients, leads to the breakdown of the DGC complex, a histological marker of the disease [41]. However, the site of the mutation determines whether these interactions are abolished in BMD patients, resulting in diverse phenotypes. For both basic research and clinical/therapeutic purposes, it is therefore of interest to establish a correlation between the genotype and the molecular and structural consequences of in-frame mutations for the encoded protein.

To this end, we have developed a new database called eDystrophin, specifically dedicated to providing information about the in-frame mutations of the *DMD* gene and their consequences for dystrophin molecules. The eDystrophin database includes both in-frame *DMD* mutations identified at a routine diagnostic laboratory for these mutations in France and published mutations. In addition to the genetic and clinical details provided by the other two available databases, the eDystrophin database provides: (1) a synthetic view of the properties of mutated dystrophin, (2) a map of modifications to binding sites for interacting protein partners; for deletions involving the central rod domain, eDystrophin provides (3) a structural model of the mutation site and (4) a specific comment indicating whether a correct filamentous 3D-structure is reconstituted around the mutation site. Finally, this new database focuses on the protein rather than the gene, providing a new vantage point regarding in-frame mutations of the *DMD* gene, with the finding that the gene exons and protein domains are “in phase” for the specific central rod domain of dystrophin. This phasing controls the ability of the internally truncated dystrophin molecules to reconstitute a hybrid repeat unit able to fold into a triple coiled-coil, resembling the native repeats present in full-length dystrophin. This database is freely available from http://edystrophin.genouest.org/, and all the information provided can be downloaded.

## Methods

eDystrophin is a relational database developed in MySQL 5.1.37 within the MAMP package [42]. The website was constructed with XHTML, PHP 5 and JavaScript. The database and the website are hosted by the BioGenouest platform [43] and run with the Apache web server, version 2.2.3. MyDomains [44] was used to represent the mutated proteins. The Jmol applet [45] is embedded for the visualization of three-dimensional structure-homology modeling.

### Database construction

#### Gene data

The *DMD* cDNA sequences for the seven known tissue-specific promoters and the positions of the 79 exons were obtained from GenBank (RefSeq in Table 1).

**Table 1:** ***DMD*** **transcript variants and their tissue expression**

**Promoters**	**Publication**	**Tissue specificity**	**Alternative splicing**	**mRNA RefSeq**	**Protein RefSeq**
dp427m	(Koenig *et al.*, 1989)[16]	Skeletal muscle, heart muscle, glial cells	Not referenced	NM_004006.2	NP_003997.1
dp427c	(Chelly *et al.*, 1990)[46]	Brain, retina	Not referenced	NM_000109.3	NP_000100.2
dp427p	(Chelly *et al.*, 1990)[46]	Purkinje cells, muscle	Not referenced	NM_004009.3	NP_004000.1
dp260	(D'Souza *et al.*, 1995)[47]	Retina	No but two alternative exon 1	1: NM_004011.3	1: NP_004002.2
				2: NM_004012.3	2: NP_004003.1
dp140	(Lidov *et al.*, 1995)[48]	Brain, kidney, retina	No splicing - > dp140	NM_004013.2	NP_004004.1
			Exons 71 & 78 - > dp140ab	NM_004022.2	NP_004013.1
			Exon 78 - > dp140b	NM_004021.2	NP_004012.1
			Exons 71 to 74 & 78 - > dp140bc	NM_004023.2	NP_004014.1
			Exons 71 to 74 - > dp140c	NM_004020.3	NP_004011.2
dp116	(Byers *et al.*, 1993)[49]	Schwann cells	Not referenced	NM_004014.2	NP_004005.1
dp71	(Hugnot *et al.*, 1992)[50]	Everywhere except skeletal muscle	No splicing - > dp71	NM_004015.2	NP_004006.1
			Exons 71 - > dp71a	NM_004017.2	NP_004008.1
			Exon 71 & 78 - > dp71ab	NM_004018.2	NP_004009.1
			Exon 78 - > dp71b	NM_004016.2	NP_004007.1
			Stop at Exon 70 - > dp40	NM_004019.2	NP_004010.1

#### Wild-type dystrophin data

**Figure 1:**
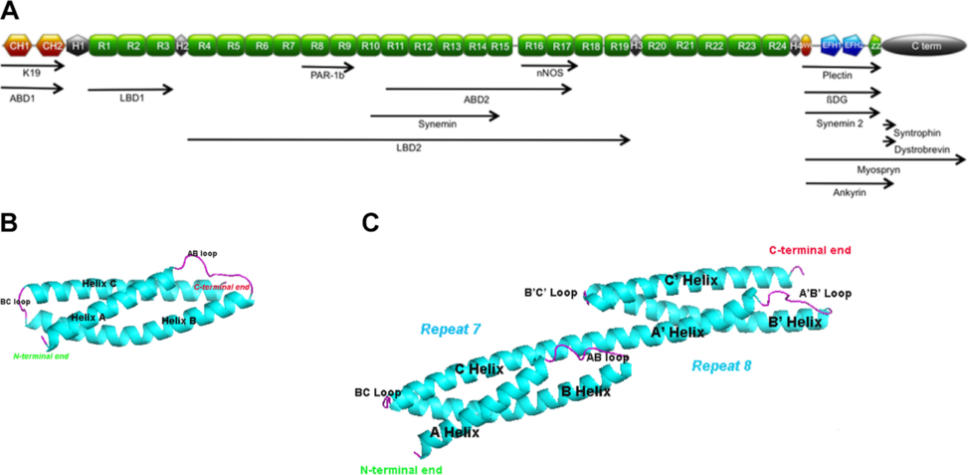
**The dystrophin molecule and its partners.** (**A**) Schematic representation of the molecule with the structural domains CH1 and CH2, constituting actin-binding domain 1, hinges 1 to 4 (H1 to H4), spectrin-like repeats 1 to 24 (R1 to R24); the WW domain and EF hand–region constituting the Cys-rich domain; ZZ, the zinc finger domain; and the C-terminal domain (C term) and its partners. (**B**) Model of the three-dimensional structure of repeat 7 folded into a triple coiled-coil, consisting of three helices, A, B, and C, joined by two loops, AB and BC. (**C**) Model of the three-dimensional structure of tandem repeats R7-8 of the rod domain. Each repeat is composed of three alpha helices folded into a triple coiled-coil: R7 (helices A, B, and C, joined by loops AB and BC) and R8 (helices A’, B’ and helix C’, joined by loops A’B’ and B’C’). A long common helical linker is formed between the two repeats by R7 helix C and R8 helix A’.

Sequence data for the 16 isoforms of wild-type dystrophin were downloaded from GenBank. Several of these isoforms are generated by alternative splicing in specific tissues (Table 1). The boundaries of the structural and functional domains were defined according to the findings of 19 published papers and three domain-search tools (Additional file 1, Table S1). This resulted in the definition of 35 structural and 15 binding domains in the eDystrophin database (Figure 1A). All of the variants of each domain mentioned by the different authors are indexed in the database (See the “Knowledge” section), together with the original publication reference.

Two X-ray crystallographic structures of dystrophin domains have been reported: one for actin-binding domain 1 at the N-terminal end [PDB: 1DXX] [51] and the other for a WW domain and EF hand–region fragment complexed with a β-dystroglycan peptide [PDB: 1EG3] [52]. We recently used homology modeling to propose the three-dimensional structure models of the 24 central rod domain repeats [53]. The database gives models for isolated repeats (Figure 1B) and for tandem repeats (Figure 1C). Each repeat consists of three α-helices: A, B, and C, and A’, B’, and C’ for the following repeat. These two structures and all the structure models can be visualized and downloaded from the eDystrophin database (from the Explore database section).

#### Data for mutated dystrophin

**Figure 2:**
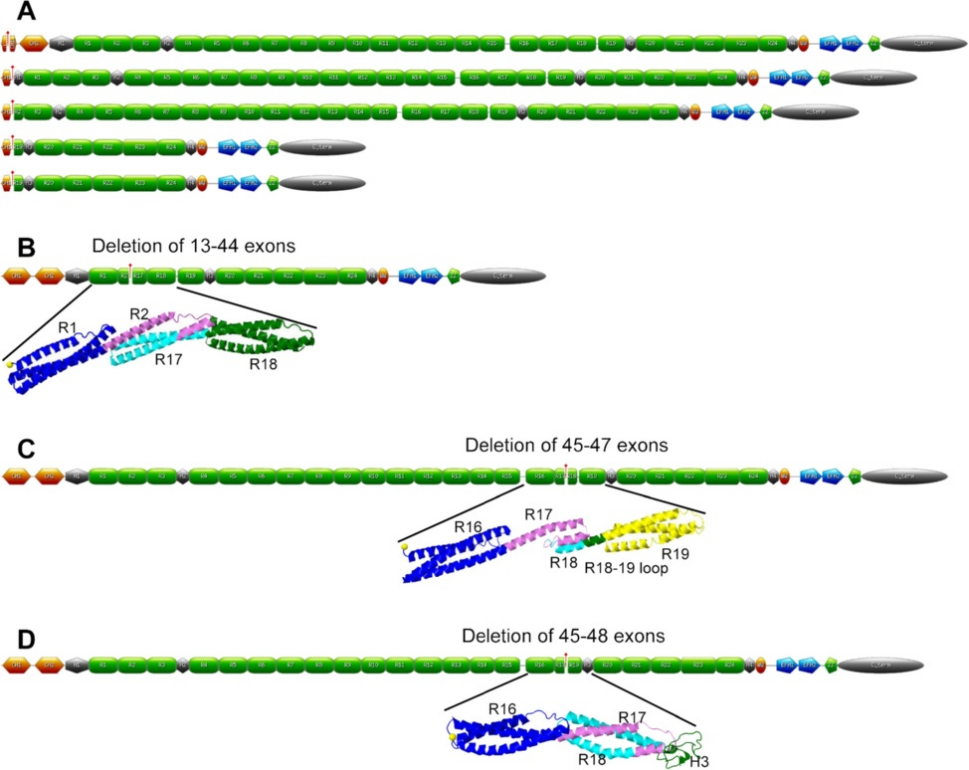
**Representation of the dystrophin proteins generated from genes with several types of deletions.** (**A**) Illustration of the proteins produced from genes with deletions of exons corresponding to the 3’ terminus encoding actin-binding domain 1. The red spot indicates the deletion site. (**B**) Deletion of exons 35 to 44: representation of the entire molecule with a tag at the deletion site and a model of the three-dimensional structure at the deletion site, showing that a hybrid repeat is reconstituted from parts of repeats 2 and 17. (**C**) Deletion of exons 45 to 47: representation of the entire molecule with a tag at the deletion site and the homology model of three-dimensional structure at the deletion site, showing that the junction between the C-terminal part of repeat 17 and the N-terminal part of repeat 18 does not allow the reconstitution of a hybrid repeat. (**D**) Deletion of exons 45 to 48: representation of the entire molecule with a tag at the deletion site and the homology model of the three-dimensional structure at the deletion site, showing that a hybrid repeat is formed from parts of repeats 17 and 18.

Our principal goal was to provide information about the structure of the protein in cases of *DMD* in-frame mutation, as a valuable tool for exon skipping therapy. We collaborated with one of the two existing databases — the French UMD-DMD database [40] — resulting in the inclusion of all patients carrying in-frame mutations from the largest French cohort, for whom detailed genetic and molecular investigations had been carried out. Data from published studies reporting well characterized exon deletions/duplications were also included. The eDystrophin database compiles 209 different in-frame mutations from a total of 945 patients. One hundred of these mutations were described in previous studies, 67 were provided by the *Laboratoire de Biochimie et Génétique Moléculaire* (LBGM, Cochin Hospital, APHP, Paris) and 42 were present in both sources (Additional file 2, Table S2). The mutations are named according to Human Genome Variation Society nomenclature recommendations [54]. For each mutation, the cDNA sequence was predicted and is available from eDystrophin (See the “Explore database” section). As mRNA studies are rarely performed in cases of exon deletion/duplication, we did not consider mRNA levels in eDystrophin. For duplications, we cannot understand events at the protein level unless we know how the duplication is arranged at the gene level. We therefore assumed that repeats were in tandem and not in opposing directions, in which case a stop codon might occur, resulting in the absence of dystrophin and a DMD phenotype. The eDystrophin database also provides some general clinical and protein information (see below) for each of the in-frame *DMD* mutations. For each of the 209 protein sequences derived from these in-frame mutations, a map of the conserved and altered structural and binding domains of dystrophin was produced, including deletions of exons at the 3’ end encoding actin-binding domain 1 (Figure 2A) and deletions of exons 13–44, 45–47 and 45–48 (Figure 2B, C, D).

For each deletion of exons encoding part of the central rod domain (exons 10–61), homology modeling was used to determine the structure of the protein at the junctions on either side of the deletion. Homology modeling was performed on the automated server, I-TASSER [55,56], as in our previous study on native repeats [53]. I-TASSER combines various techniques, including threading, *ab initio* modeling and structure-refinement approaches, for the prediction of protein structures. For each submitted sequence, one to five models are produced based on homology to the spectrin repeats, the crystallographic structures of which have been resolved. Briefly, the C-score in I-TASSER estimates the quality of the predicted model, based on the significance of the threading alignments and the convergence of the simulations. C-score values typically lie in the range of −5 to 2, with higher scores indicative of a better model. Only the model with the best C-score is retained in the eDystrophin database. Furthermore, three-dimensional structure models of isolated hinges were submitted to I-TASSER and were incorporated into models as necessary. These new structures, made of separated blocks, were minimized twice, in water and 50mM NaCl. The models were further analyzed by graphical display with PyMOL [57] and evaluated with PROCHECK (to check the stereochemistry information supplied by ProSA-web [58,59]) and Verify3D [60,61] (for structure validation). Ramachandran plots showing the amino-acid distribution are provided by eDystrophin, indicating the energetically favorable regions for peptide bond torsion angles for each amino-acid of the protein. The structural model is considered of higher quality if the distribution of amino-acid torsions is restricted to the regions allowed in the plot.

The models can be visualized online with Jmol [45], using a color code for each region of the protein. Comparison of the structure of the mutated protein with native repeat folding is provided by eDystrophin, through a static view of a native three-repeat fragment of dystrophin in a parallel box. Static images and PDB files for the wild-type and mutated models can be downloaded.

#### Clinical data

In total, 945 clinical records (531 provided by the LBGM and 414 from published studies) were included in eDystrophin. The database provides a brief description of the disease phenotype corresponding to the most commonly used clinical classification. The DMD and BMD phenotypes are attributed to cases in which the patient loses the ability to walk before the age of 13 years and after the age of 16 years, respectively. The intermediate muscular dystrophy (IMD) phenotype is used to describe patients who stopped walking between the ages of 13 and 16 years. The DCM phenotype is attributed to the subgroup of patients with isolated cardiomyopathy without skeletal muscle involvement, whereas the “pending” subgroup corresponds to patients with insufficient clinical data for correct classification of their phenotype. Finally, the “asymptomatic” phenotype is assigned to patients with no myopathic or cardiac symptoms at their most recent check-up.

The results of dystrophin immunostaining and/or western blot studies with three specific antibodies against different domains of dystrophin were available for 360 patients. The Dys-1 antibody is specific to repeats 8–9 in the central rod region; the Dys-2 antibody is specific to the C-terminus (residues 3669–3685); and the Dys-3 antibody is specific to the N-terminus (residues 321–494). For the LBGM patients, data from immunostaining studies are presented according to staining regularity (normal, regular, irregular, mosaic, no signal with revertant fibers, and no signal) and intensity (high, medium, and low). Western blots with the same three antibodies were used to assess the quantity (high, medium or low) and size (increased, normal, reduced or undetected) of the three major regions of the mutated protein. Thus, for each of the recorded in-frame mutations, the eDystrophin database displays the percentage of patients carrying the mutation with resulting in the production of low, normal or high levels of dystrophin proteins of small, normal or large size. In addition, for cases in which clinical data are available, the presence of cardiomyopathy or mental retardation at the last clinical examination is also displayed. The aim of eDystrophin was not to provide detailed descriptions of clinical symptoms, dystrophin immunofluorescence and western blot status for each patient, but a global view for each subgroup of patients carrying the same in-frame *DMD* mutation.

## Results

### Website organization

The eDystrophin website contains four distinct sections: “*Knowledge*”, “*Explore database*”, “*Statistics*” and “*Links*”. The “*Knowledge*” pages provide background information about the dystrophin gene and protein, the diseases associated with mutations of the gene and current cell- and gene-therapy strategies. The “*Explore database*” pages contain data that can be downloaded, and this section is divided into two parts: ‘*Wild-type dystrophin*’, and ‘*Mutated dystrophin*’. The “*Statistics*” section provides a brief summary of statistics for eDystrophin content. Finally, the “*Links*” section provides some useful links and a list of previous publications reporting well characterized exon deletions/duplications implemented in eDystrophin.

The ‘*Wild-type dystrophin*’ pages provide all of the wild-type sequences and the corresponding three-dimensional structures of the domains. The cDNA and protein sequences of the 16 isoforms can be obtained by clicking on the “Isoform full-length sequences” tab. A diagram of the organization of the cDNA can be obtained by clicking on the “Exon sequences” tab, and the sequences of the 79 exons can be obtained by clicking on the chosen exon. The sequences of the various domains, with all of the versions reported in published studies, can be obtained by clicking on the “Structural domain sequences” tab, and a diagram of dystrophin with the functional domains reported in published studies can be obtained by clicking on the “Binding domain sequences” tab. By clicking on the domain, the user can download the sequence. Similarly, the three-dimensional structures found in PDB are available via the “3D-structure models” tab, as are the homology-based models of all single and tandem repeats, which can be visualized with the Jmol vizualization tool [45].

The “*Mutated dystrophin*” pages are dedicated to the 209 in-frame mutations of the human *DMD* gene included in eDystrophin. It is possible to search for mutations according to mutation type, phenotype or the domain involved.

**Figure 3:**
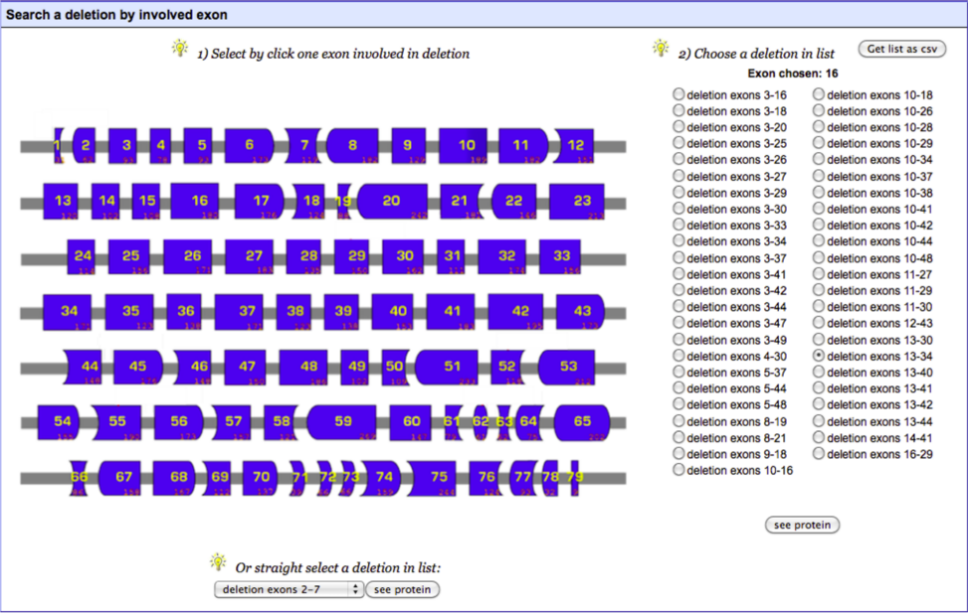
**Screenshot of “Search by mutation type/Deletions” tool.** The user can select an exon and then a deletion involving this exon. On the right side of the page, the user can select a mutation of interest from a list, to obtain data about this mutation. The user can also save this list as a csv-format file.

The “Search by mutation type” tool allows the user to search for deletions, duplications or substitutions. If “Deletions” or “Duplications” are selected, a diagram of the exons of the *DMD* gene is displayed (as in Figure 3). This allows the user to select an exon, for which a list of all deletions or duplications affecting this exon can then be obtained, or to select a mutation of interest from the list at the bottom of the page. If “Substitutions” is selected, a list of mutations is provided. The “Search by phenotype” tool provides a list of mutations “affecting at least one patient” or “affecting no patient” with a specific phenotype. These phenotypes include pending or asymptomatic, BMD, IMD, DMD or DCM. The “Search by involved domain” tool provides a representation of the dystrophin protein, together with its known partners. The names of the structural domains and partners of dystrophin are active buttons. Clicking on these buttons brings up a list of mutations affecting the chosen domain or partner. All of these mutation lists are easy to save down as a csv-formatted data file.

**Figure 4:**
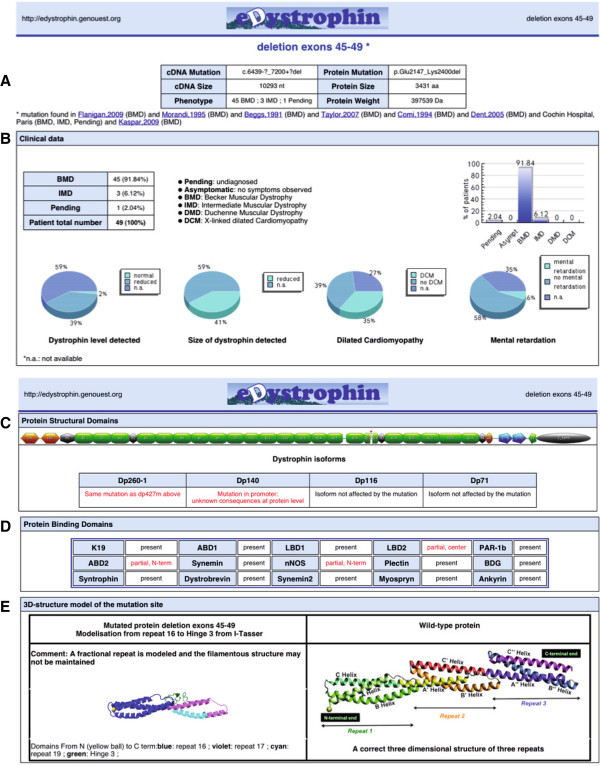
**Screenshot of data available for the deletion of exons 45 to 49.** (**A**) A general view of the webpage after loading. Description of the mutation at the nucleotide and protein levels, cDNA and protein size, the molecular weight of the mutated protein, a link to cDNA and protein sequences and a list of references reporting patients carrying the mutation are available in table form. Detailed information about clinical data, structural and binding domains and models of three-dimensional structure can be obtained by clicking on the boxes below. (**B**) The “*Clinical data*” tab on the first line provides access to a table showing the distribution of phenotypes. In the second line, pie-charts showing the amount and size of dystrophin, as determined by western blotting, the presence of cardiomyopathy and mental retardation are given. (**C**) The “*Structural domains*” tab provides a schematic representation of the mutated dystrophin. The sequences of each protein domain are available. (**D**) The “*Binding domains*” tab indicates, in red, the changes to the binding domains caused by the mutations listed in the table. (**E**) The “3D-structure model of the mutation site*”* tab shows the model of the three-dimensional structure of the domains around the mutation junctions (here R16, R17, R19 and H3). The secondary structure predicted by I-TASSER is displayed above the model. The PDB file and the Ramachandran plot are also available. The modeling tab is available only for the deletion of exons encoding part of the central rod domain. All information can be saved down in the form of PDF files.

Regardless of the way in which the list of mutations was obtained, clicking on the “see protein” button opens a new page showing a summary table with a global view of the effects of the mutations and four horizontal tabs displaying details (Figure 4A). By clicking on the ‘Clinical data’ tab, the user can obtain the number of patients listed in the database carrying a given mutation, and information about the overall distribution of phenotypes, the severity of the disease in BMD patients, the presence or absence of cardiomyopathy and mental retardation and the size and amount of dystrophin (when such data are available) (Figure 4B).

Clicking on the “Protein structural domains” tab provides a map of the protein domains modified by the mutation and a table summarizing the consequences of the deletion for the four shorter dystrophin isoforms (Figure 4C). Similarly, clicking on the “Protein binding domains” tab brings up a map of modifications to the binding domains of the mutated protein (Figure 4D). Finally, clicking on the “3D-structure model of the mutation site” tab provides the user with a model of the three-dimensional structure of the regions on either side of the mutation in cases in which exons encoding part of the central rod domain are deleted (Figure 4E). The user can freely download a PDF summary file including the clinical data described above, information about structural and binding domains and the three-dimensional structure model. A final comment about the impact of the mutation on the filamentous structure of the protein is also provided.

### Overview of the content of the database

#### Statistics

**Figure 5:**
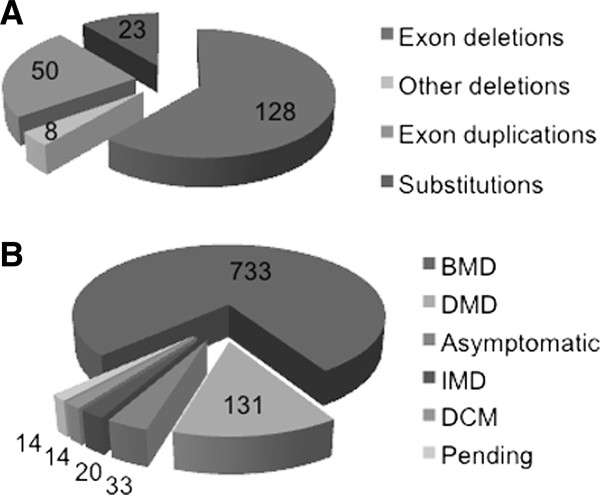
**Statistics for mutations recorded in the eDystrophin database.** (**A**) Mutation types: the number of cases is shown for each of 209 different mutations. (**B**) Phenotype distribution: for each phenotype, the number of patients concerned, from a total of 945 patients, is shown.

The 209 mutations recorded in the eDystrophin database include 128 large deletions of one or several exons (61% of the mutations listed) and 50 large duplications of one or several exons (24% of the mutations). There are also 23 missense mutations (11%), and 8 small in-frame deletions (4%) (Figure 5A).

Figure 5B shows the phenotype distribution for all the patients. Of the 945 patients, 733 (78%) had the BMD phenotype, 131 (14%) had the DMD phenotype (most of these patients had mutations towards the 3’ end of the *DMD* gene), and 20 patients (2%) had the IMD phenotype. Thus, 16% of the patients registered in the eDystrophin database are exceptions to the Monaco rule [26], as they carry in-frame mutations associated with the severe DMD/IMD phenotypes. Deletions of exons 60 to 79 are generally observed only in DMD patients, but deletions of exons 45–79 and of exon 60 have been reported in at least one BMD patient (Additional file 3 Figure S1A, B). This is consistent with previous reports showing that deletions involving the Cys-rich domain are more deleterious than mutations involving the central rod domain. Similarly, 18 mutations starting at exon 3 have been found in at least one DMD patient, whereas only nine such mutations have been found in at least one BMD patient. As previously reported, deletions affecting ABD1 were generally found to be more deleterious than those affecting the central domain [23,40]. Twenty-nine of the deletions involving the central domain were found in at least one DMD patient, whereas 60 were found in at least one BMD patient. Thirty-one of the duplication mutations were observed in at least one DMD patient (Additional file 3, Figure S1C,D), and 24 were observed in at least one BMD patient. These findings indicate that duplications are generally more deleterious than deletions.

#### Case studies

We illustrate the use of the eDystrophin database for analysis of the consequences of a specific in-frame *DMD* mutation, by studying two exon deletions: deletion of exons 13–44 (c.1483-? 6438 + ?del; p.Val495_Lys2146del) which is a proximal large deletion with a low frequency, and deletion of exons 45–47 (c.6439-?_6912 + ?del; p.Glu2147_Lys2304del), a very frequent, distal, relatively short deletion. The easiest way to proceed is to select one of the mutated exons from the “Mutated dystrophin” page and then to click on the “Search by mutation type”/“deletions” tabs. The desired mutation is chosen and the “see protein” tab is clicked to view the results page.

As shown in the summary at the top of the new page, the deletion of exons 13–44 has been observed in only one BMD patient (“Clinical data” tab). This deletion leads to the production of a 2033-residue dystrophin protein with a molecular weight of 234kDa, which is smaller than its wild-type counterpart (3685 residues and a molecular weight of 427kDa) and was originally reported in [22]. All the data on the page can be downloaded as a pdf file, by clicking on “Get pdf file”. The “Protein structural domains” tab shows that the region from the C-terminal part of repeat 2 to the N-terminal part of repeat 17 (including hinge 2), is missing from the mutated protein (Figure 2B). Furthermore, this deletion affects the promoter of the Dp260 and Dp140 isoforms but has no effect on Dp116 and Dp71. The “Protein binding domains” tab shows that the deletion encompasses the entire PAR-1b and synemin-binding domains and partly modifies the LBD1, LBD2, ABD2, and nNOS binding domains. Clicking on the “3D-structure model of the mutation site” tab brings up the I-TASSER-built three-dimension structural model, showing that a long helix is reconstituted at the mutation site junction between the N-terminal part of repeat 2 and the C-terminal part of repeat 17 (Figure 2B). Thus, the reconstitution of a triple coiled-coil, as in the wild-type three-repeat model, may occur. This I-TASSER model has a C-score of 0.66, indicating a correct fold, as confirmed by Verify3D, ProSA-web and Procheck, all of which indicated a high overall quality for the model. The Ramachandran plot showed that 91.7% of the residues were in the most favored regions, with 1.7% of residues in disallowed regions. These disallowed residues are located in loops, which are often poorly defined. These values are compatible with a crystal structure. As shown in the box, we can therefore conclude that a correct filamentous three-dimensional structure, in the form of a hybrid repeat generated by the concatenation of two truncated repeats, is reconstituted at the new junction. Such a hybrid repeat was hypothesized for the deletion of exon 41–42 in an *in vitro* experiment. The author of the study concerned concluded that this hybrid repeat is viable and has some of the properties of the native repeat [62].

The second case study is that of the deletion of exons 45–47. Unlike the first example, this deletion is very frequent, having been observed in 223 patients (23.6% of the eDystrophin cohort). The resulting protein is 3527 residues long, with a calculated molecular weight of 409kDa. The “Clinical data” tab shows that the observed phenotypes correspond to BMD in 96% of patients, associated cardiomyopathy in 19% of patients, and features suggesting mental retardation in 2% of patients. Dystrophin protein levels were reported to be lower than normal in 30% of the patients and normal or high in 1% of patients, with no data available for the remaining patients. The protein was small in 30% of patients and of normal size in 3% of patients, with no data available for the remaining patients. Thus, data concerning the amount and size of the dystrophin protein are missing for 67% of the patients. The “Protein structural domains” tab shows that the deletion eliminates the C-terminal end of repeat 17 and the N-terminal end of repeat 18 (Figure 2C). Dp260 is deleted, along with Dp427m and the Dp140 promoter is affected by the deletion, but the wild-type Dp116 and Dp71 isoforms are unaffected. The “Protein binding domains” tab shows that the ABD2, LBD2, and nNOS binding domains are partially modified by this deletion. As can be seen by clicking on the “3D-structure model of the mutation site” tab, the junction region on either side of the mutation is correctly folded, consistent with the findings of Verify 3D and ProSA-web analysis. The Ramachandran plot shows that 90.2% of the residues are in the most favored regions, and 1.6% residues are in disallowed regions, specifically in loops between helices. However, the model also includes small helices that do not reconstitute a triple coiled-coil (Figure 2C). This implies that no hybrid repeat reconstitution occurs at the junction, with a fractional repeat formed instead and resulting in an incorrect filamentous 3D structure. The term “fractional repeat” has previously been used to describe the joining of two truncated repeats without the reconstitution of a triple coiled-coil similar to that observed in native repeats [62,63]. This conclusion is again displayed in the box.

### Exon phasing versus repeat phasing

The triple coiled-coil structure of the wild-type dystrophin repeats requires the amino-acid sequences to have a seven-residue pattern (the heptad), with apolar residues located alternately three and four residues apart. The α-helices assemble such that they are tilted and coiled around one another, each in an opposite direction to the other helices. This generates the multi-stranded structure of the coiled-coil [64,65]. Such structures may be formed from two or three helices. Like all spectrin-like repeats, dystrophin repeats consist of three helices. As shown above, exon deletions may or may not allow the reconstitution of a triple coiled-coil at the junction between the sequences on either side of the deletion.

**Figure 6:**
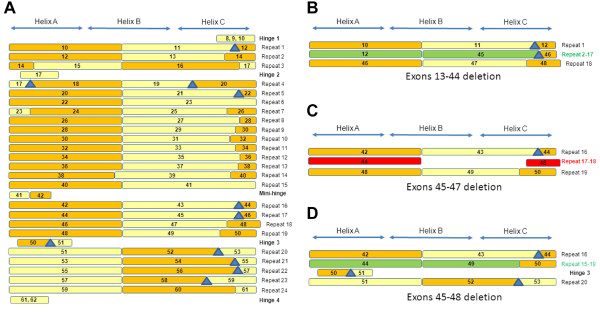
**Fusion of the exon borders and repeat alignment.** (**A**) The 24 repeats are represented by the exons encoding them (not by their helices) as rectangles with the following color code: orange for even-numbered exons and light yellow for odd-numbered exons. The frameshifting exon borders are shown as blue triangles. The approximate position of the helices is indicated above the figure. (**B**) Focus on the deletion site for the deletion of exons 13 to 44, showing the reconstitution of a hybrid repeat by the joining of exons 12 and 45 (in green), maintaining the phasing of exon coding for a reconstituted B helix. (**C**) Focus on the deletion site for the deletion of exons 45 to 47, showing how the joining of exons 44 and 48 (in red) does not respect the phasing of the repeats and the presence of an extra sequence inconsistent with a repeat. (**D**) Focus on the deletion site for the deletion of exons 45 to 48, showing how the hybrid repeat can be reconstituted by the joining of exons 44 and 49 (in green), maintaining the phasing of the exons encoding a reconstituted B helix

Comparison of the heptad pattern of the repeat alignment obtained by Winder [7] with the exon boundaries showed that all the B helices were encoded by two successive in-frame coding exons (Additional file 3, Figure S1). A simplified diagram of this global organization of exons and repeats in the central rod domain of dystrophin is shown in Figure 6A. Each line represents a repeat or a hinge. The exons encoding the repeats are represented by rectangles, with alternating colors for clarity: even-numbered exons are shown in orange and odd-numbered exons are shown in light yellow. In all the repeats other than repeat 14, the B helices appear to be encoded by two successive in-frame exons with the boundaries precisely aligned with the third heptad of the B helices (Additional file 4, Figure S2).

Thus, an in-frame deletion of two successive exons, the first of which encodes the C-terminal part of a B helix, would be expected to result in deletion of the end of this B helix and of the C helix of the first repeat, together with the beginning of the B helix of the following repeat. This deletion results in a concatenation of the first part of the B helix of the first repeat with the second part of the B helix of the second repeat. Consequently, the heptad pattern remains similar to that in wild-type dystrophin, and the domain can fold like a native repeat, thereby constituting a hybrid repeat. This observation confirms the previous report of an observation of a hybrid repeat [62]. A similar phenomenon may occur for many deletions involving two such exons or multiples of two exons. In situations in which the formation of a hybrid repeat is not possible, fractional repeats, in which the heptad pattern of alignment is not respected at the new junction between the sequences on either side of the deletion, may form, as previously suggested [63].

## Discussion

The eDystrophin database is a new biomedical resource for clinicians and researchers working on human dystrophin diseases. This dedicated database for the dystrophin protein specifically aims to provide information about in-frame *DMD* mutations and their consequences for the dystrophin protein. It provides a framework for the analysis of such mutations, by presenting a large body of information for both wild-type and mutated dystrophin proteins, including findings relating to the structure of these proteins and their interactions with known partners. Although eDystrophin is a locus-specific database, it was not constructed with an existing database system, such as LOVD [66,67] or UMD [68,69]. Indeed, such systems are more useful for DNA variant databases and are not suitable for the construction of a protein-based database like eDystrophin.

In human dystrophin diseases, the ratio of the frequency of DMD to that of BMD is approximately 2/3 – 1/3 [40,70]. Most cases of DMD are caused by frame-shift mutations, whereas BMD is generally caused by in-frame mutations, although exceptions have been reported [26]. Documented cases of in-frame mutations are largely underrepresented in existing databases, and the primary aim of the eDystrophin database project was to redress the balance, by developing a dedicated information source for in-frame mutations. Unlike frame-shift mutations, in-frame mutations lead to the production of proteins with various degrees of functionality. The secondary goal of eDystrophin was therefore to determine and show the predicted consequences of these mutations for the composition and structure of the encoded proteins and their clinical consequences. In this first version of eDystrophin, patients and in-frame mutations were obtained from one of the major French contributors to the UMD-DMD database and from published studies. Evidently, the database could be expanded in the near future by including mutations and patients from around the world, which would probably yield more accurate phenotype-genotype correlations.

However, dystrophin is a large protein, and it is a challenge to investigate the consequences of mutations of its gene. The dystrophin protein has two principal roles: as a scaffolding protein for several interacting partners and as a filamentous protein with a mechanical and structural function, providing resistance to the stress of muscle contraction [8,13]. Any mutation altering the structure of dystrophin may therefore affect both these functions (and potentially other minor functions of the protein as well) simultaneously. Our database provides an overview of the effects of mutations on protein function. In particular, it provides the user with information about changes to interactions and about the maintenance or disruption of the filamentous structure of the mutated dystrophin protein.

Several binding partners of dystrophin have been identified, and the eDystrophin database infers changes to their binding to a mutated dystrophin variant by considering whether the interacting domains remain intact and unmodified. Based on these inferences and previous observations, deletions affecting the Cys-rich or ABD1 domains appear to be much more deleterious than those affecting the central domain [23,40]. However, we detected several mutations affecting the central rod domain and causing a DMD phenotype in a substantial number of patients. In these patients, mRNA levels may have been low and/or unstable, accounting for the presence of little or no protein. Careful re-examination of the boundaries of the mutation is also necessary for these patients. Indeed, Taylor *et al.* (2007, PhD thesis) re-examined a large cohort of DMD patients with in-frame deletions affecting the central rod domain and found that most were frame-shift mutations, consistent with the Monaco reading frame rule. Furthermore, we cannot entirely exclude the possibility of two mutations occurring in the same gene. For the other DMD patients carrying in-frame mutations, uncertainties remain concerning the levels or stability of the corresponding mRNA.

We obtained models of the three-dimensional structure of the new junctions created between the sequences on either side of the deletions in the central rod domain, as previously described [53]. The database provides a computational model for each in-frame deletion collected. An analysis of the structural features of these new junctions showed that two outcomes were possible: the reconstitution of a hybrid repeat and the formation of a fractional repeat in situations in which it was not possible to form a hybrid repeat. The likelihood of hybrid repeat formation depends on the phasing of the exon boundaries with the center of the B helix of the repeats. The reconstitution of a hybrid repeat can be assumed to occur because the major factor controlling this folding pattern is the presence of a heptad pattern. As this pattern is respected in cases in which the deletion creates a new junction between the first half of one B helix and the second half of the next, from two truncated repeats, coiled-coil folding similar to that in native repeats would be expected [64,65]. By contrast, in fractional repeats, the α-helices can fold correctly, but the heptad pattern is not respected and a three-dimensional coiled-coil structure therefore cannot be obtained. This may result in a less stable deletion site than for native and hybrid repeats. The hypothesis that repeats phasing in truncated dystrophins is essential to ensure a high level of protein function has already been tested. Transgenic *mdx* mice were produced with several types of truncated dystrophin, some with correct and others with incorrect phasing of the repeats. However, in this previous study, native repeats were either entirely conserved or entirely lost [71]. These findings led to the “mini-dystrophin” concept for DeltaH2-R19, in which the rod domain was decreased in size by a deletion encompassing the amino acids from hinge 2 to repeat 19. By contrast the “micro-dystrophin” DeltaR4-R23 had a deletion extending from repeat 4 to repeat 23. Constructs encoding these proteins proved to be among the best therapeutic constructs for *mdx* mouse rescue. In BMD patients, phasing is not as described in these previous experiments and only hybrid repeats may be reconstituted. However, the demonstration of beneficial effects of phasing in the *mdx* mouse suggests that the presence of hybrid repeats may be associated with a milder phenotype than the presence of fractional repeats [62,63]. Such a correlation between the structural features of mutated dystrophin and clinical severity in a cohort of BMD patients has been reported for cardiomyopathy [24]. The authors constructed models of the mutated dystrophin for deletions involving exons 45 to 49 and investigated the phasing of spectrin repeats. They concluded that the absence of hinge 3 delayed the onset of dilated cardiomyopathy.

However, it should be stressed that the presence of a hybrid repeat does not itself imply a better conservation of dystrophin function than the presence of a fractional repeat. Indeed, mRNA instability or changes to protein-protein interactions may also affect the function of the mutated dystrophin, and it is not currently possible to predict these effects. Investigations of the correlation between the presence of a hybrid repeat and the severity of clinical symptoms are now required. However, the eDystrophin database can be used as a predictive tool for exon skipping–based therapy. The choice of the exon to be deleted to restore the reading frame could be based on careful consideration of the likelihood of reconstituting a hybrid repeat.

## Conclusions

The eDystrophin database is a new tool providing an overview of the proteins generated by *DMD* genes carrying in-frame mutations. It provides information about the consequences of these mutations for protein production and folding and for phenotype-genotype correlations. This database, through these features, is thus a valuable tool for predicting the efficacy of exon-skipping therapy for DMD patients.

### Supporting data

The supporting datasets are provided within the article and the additional files.

## Competing interests

The authors declare that they have no competing interests.

## Authors’ contributions

ELR, FBH, RBY and JC initiated and supervised the project. AN, CLM and FBH created the database. AN, RBY, FL and ELR monitored data collection. AN and ELR created and analyzed the structural models. ALL authors participated in the writing of the manuscript and approved its submission.

## Author’s information

Frédérique Barloy-Hubler: IFR GFAS - http://ifr140.univ-rennes1.fr/plates-formes/Amadeus/.

## Supplementary Material

**Additional file 1 Table S1.** **Additional file 1 Table S1.** provides the references from which information about the structural and binding domains of dystrophin described in eDystrophin was obtained [7,51,52,72-90]. Click here for file

**Additional file 2 Table S2.** **Additional file 2 Table S2.** provides the origin of the mutations described in the eDystrophin database [18,20,22,24,91-102]. Click here for file

**Additional file 3 Figure S1.** **Additional file 3 Figure S1.** Statistics for mutations included in eDystrophin. Exon deletions (A, B) and duplications (C, D) associated with at least one DMD (A, C) or one BMD (B, D) patient. Each line represents a type of exon deletion (A and B) or duplication (C and D). Click here for file

**Additional file 4 Figure S2.** **Additional file 4 Figure S2.** Sequence alignment of the 24 spectrin-like repeats of dystrophin. Repeats were aligned by ClustalW, using default parameters, as described by Winder *et al.*[7]. The alignment was visualized in Jalview. In the first line, heptad motifs are indicated, showing the hydrophobic residues in the (a) and (d) positions. The repeat numbers and the number of residues per repeat are indicated at the start of the repeat sequence. The presence of absence of hinges 2 and 3 is indicated in separate lines. There are two extra sequences at the ends of repeats 15 and 18 not aligned with the heptad pattern. The presence of these sequences is indicated at the end of the corresponding lines. At the end of each line, the numbers of the exons encoding the repeat are indicated in parentheses. The repeat sequences are highlighted alternately in orange (even-numbered exons) and light yellow (odd-numbered exons). The rectangle indicates the middle of the B helices of the repeats. If the successive exons are not in frame, the residues are shown in red. The alignment reported by Koenig *et al.*[76] is also mentioned: the residues at the start of the repeats are underlined when they differ from Winder’s alignment. Click here for file
