# Supplementary material for: Assessment of the structural and functional impact of in-frame mutations of the DMD gene, using the tools included in the eDystrophin online database
Source: Orphanet J Rare Dis. 2012 Jul 9;7:45. doi: 10.1186/1750-1172-7-45 (PMC3748829; doi:10.1186/1750-1172-7-45)
Supplement: Additional file 1 Table S1. — provides the references from which information about the structural and binding domains of dystrophin described in eDystrophin was obtained [7,51,52,72-90]. [file 1750-1172-7-45-S1.docx]

**Additional file 1: table S1.** References from which the structural and binding domains of dystrophin described in eDystrophin are issued.

| **Structural domains** | **References** |
| --- | --- |
| CH domain 1 | (Norwood et al., 2000) [51], (Banuelos et al., 1998) [72], cd [73], smart [74], pfam [75] |
| CH domain 2 | (Norwood et al., 2000) [51], (Banuelos et al., 1998) [72], cd [73], smart [74], pfam [75] |
| Hinge 1 | (Koenig and Kunkel, 1990) [76] |
| repeat 1 | (Koenig and Kunkel, 1990) [76], (Winder et al., 1995) [7] |
| repeat 2 | (Koenig and Kunkel, 1990) [76], (Winder et al., 1995) [7] |
| repeat 3 | (Koenig and Kunkel, 1990) [76], (Winder et al., 1995) [7] |
| Hinge 2 | (Koenig and Kunkel, 1990) [76], (Winder et al., 1995) [7] |
| repeat 4 | (Koenig and Kunkel, 1990) [76], (Winder et al., 1995) [7] |
| repeat 5 | (Koenig and Kunkel, 1990) [76], (Winder et al., 1995) [7] |
| repeat 6 | (Koenig and Kunkel, 1990) [76], (Winder et al., 1995) [7] |
| repeat 7 | (Koenig and Kunkel, 1990) [76], (Winder et al., 1995) [7] |
| repeat 8 | (Koenig and Kunkel, 1990) [76], (Winder et al., 1995) [7] |
| repeat 9 | (Koenig and Kunkel, 1990) [76], (Winder et al., 1995) [7] |
| repeat 10 | (Koenig and Kunkel, 1990) [76], (Winder et al., 1995) [7] |
| repeat 11 | (Koenig and Kunkel, 1990) [76], (Winder et al., 1995) [7] |
| repeat 12 | (Koenig and Kunkel, 1990) [76], (Winder et al., 1995) [7] |
| repeat 13 | (Koenig and Kunkel, 1990) [76], (Winder et al., 1995) [7] |
| repeat 14 | (Koenig and Kunkel, 1990) [76], (Winder et al., 1995) [7] |
| repeat 15 | (Koenig and Kunkel, 1990) [76], (Winder et al., 1995) [7] |
| repeat 16 | (Koenig and Kunkel, 1990) [76], (Winder et al., 1995) [7] |
| repeat 17 | (Koenig and Kunkel, 1990) [76], (Winder et al., 1995) [7] |
| repeat 18 | (Koenig and Kunkel, 1990) [76], (Winder et al., 1995) [7] |
| repeat 19 | (Koenig and Kunkel, 1990) [76], (Winder et al., 1995) [7] |
| Hinge 3 | (Koenig and Kunkel, 1990) [76], (Winder et al., 1995) [7] |
| repeat 20 | (Koenig and Kunkel, 1990) [76], (Winder et al., 1995) [7] |
| repeat 21 | (Koenig and Kunkel, 1990) [76], (Winder et al., 1995) [7] |
| repeat 22 | (Koenig and Kunkel, 1990) [76], (Winder et al., 1995) [7] |
| repeat 23 | (Koenig and Kunkel, 1990) [76], (Winder et al., 1995) [7] |
| repeat 24 | (Koenig and Kunkel, 1990) [76], (Winder et al., 1995) [7] |
| Hinge 4 | (Koenig and Kunkel, 1990) [76] |
| EF hand 1 | pfam [75], (Huang et al.,) [52], (Ishikawa-Sakurai et al., 2004) [77] |
| EF hand 2 | (Huang et al.,) 52], (Ishikawa-Sakurai et al., 2004) [77], pfam [75] |
| Zinc Finger | (Ishikawa-Sakurai et al., 2004) [77], pfam [75], (Hnia et al., 2007) [78], smart [74], cd [73], (Hnia et al., 2007) [78] |
| C term | (Koenig et al., 1988) [79] |

| **Binding domains** | **References** |
| --- | --- |
| K19 | (Stone et al., 2005) [80] |
| ABD1 | (Norwood et al., 2000) [51], (Banuelos et al., 1998) [72] |
| LBD1 | (Legardinier et al., 2009) [81] |
| LBD2 | (Legardinier et al., 2009) [81] |
| PAR-1b | (Yamashita et al., 2010) [82] |
| ABD2 | (Amann et al., 1998) [83] |
| Synemin | (Bhosle et al., 2006) [84] |
| nNOS | (Lai et al., 2009) [85] |
| Plectin | (Rezniczek et al. 2007) [86] |
| BDG | (Ishikawa-Sakurai et al., 2004) [77], (Huang et al.,) [52] |
| Syntrophin | (Newey et al., 2000) [87] |
| Dystrobrevin | (Sadoulet-Puccio et al., 1997) [88] |
| Synemin2 | (Bosle et al., 2006) [84] |
| Myospryn | (Reynolds et al., 2008) [89] |
| Ankyrin | (Ayalon et al., 2008) [90] |
