# Supplementary material for: Assessment of the structural and functional impact of in-frame mutations of the DMD gene, using the tools included in the eDystrophin online database
Source: Orphanet J Rare Dis. 2012 Jul 9;7:45. doi: 10.1186/1750-1172-7-45 (PMC3748829; doi:10.1186/1750-1172-7-45)
Supplement: Additional file 2 Table S2. — provides the origin of the mutations described in the eDystrophin database [18,20,22,24,91-102]. [file 1750-1172-7-45-S2.docx]

**Additional file 2: table S2.** Origin of the mutations described in eDystrophin database

| **Mutations** | **Mutations (official nomenclature)** | **References** |
| --- | --- | --- |
| deletion exons 2-7 | c.32-?_649+?del | (Kaspar et al., 2009) [24], LBGM, Paris*, (Beggs et al., 1991) [18] |
| deletion exon 3 | c.94-?_186+?del | (Flanigan et al., 2009) [91], (Beggs et al., 1991) [18], (Taylor et al., 2007) [92], (Kaspar et al., 2009) [24], (Dent et al., 2005) [93] |
| deletion exons 3-4 | c.94-?_264+?del | LBGM, Paris*, (Comi et al., 1994) [20], (Kaspar et al., 2009) [24], (Taylor et al., 2007) [92], (Dent et al., 2005) [93] |
| deletion exons 3-5 | c.94-?_357+?del | LBGM, Paris* |
| deletion exons 3-9 | c.94-?_960+?del | LBGM, Paris*, (Taylor et al., 2007) [92], (Kaspar et al., 2009) [24] |
| deletion exons 3-12 | c.94-?_1482+?del | LBGM, Paris*, (Flanigan et al., 2009) [91] |
| deletion exons 3-13 | c.94-?_1602+?del | LBGM, Paris* |
| deletion exons 3-15 | c.94-?_1812+?del | (Flanigan et al., 2009) [91], LBGM, Paris* |
| deletion exons 3-16 | c.94-?_1992+?del | LBGM, Paris*, (Dent et al., 2005) [93], (Comi et al., 1994) [20] |
| deletion exons 3-18 | c.94-?_2292+?del | LBGM, Paris* |
| deletion exons 3-20 | c.94-?_2622+?del | LBGM, Paris*, (Flanigan et al., 2009) [91], (Dent et al., 2005) [93] |
| deletion exons 3-25 | c.94-?_3432+?del | (Flanigan et al., 2009) [91], LBGM, Paris* |
| deletion exons 3-26 | c.94-?_3603+?del | (Taylor et al., 2009) [94] |
| deletion exons 3-27 | c.94-?_3786+?del | (Flanigan et al., 2009) [91] |
| deletion exons 3-29 | c.94-?_4071+?del | LBGM, Paris** |
| deletion exons 3-30 | c.94-?_4233+?del | (Flanigan et al., 2009) [91] |
| deletion exons 3-33 | c.94-?_4674+?del | LBGM, Paris* |
| deletion exons 3-34 | c.94-?_4845+?del | LBGM, Paris*, (Taylor et al., 2007) [92] |
| deletion exons 3-37 | c.94-?_5325+?del | (Flanigan et al., 2009) [91] |
| deletion exons 3-41 | c.94-?_5922+?del | (Nevo et al., 2003) [95], LBGM, Paris* |
| deletion exons 3-42 | c.94-?_6117+?del | (Flanigan et al., 2009) [91] |
| deletion exons 3-44 | c.94-?_6438+?del | LBGM, Paris** |
| deletion exons 3-47 | c.94-?_6912+?del | (Taylor et al., 2007) [92] |
| deletion exons 3-49 | c.94-?_7200+?del | LBGM, Paris* |
| deletion exons 4-12 | c.187-?_1482+?del | LBGM, Paris* |
| deletion exons 4-30 | c.187-?_4233+?del | (Flanigan et al., 2009) [91] |
| deletion exon 5 | c.265-?_357+?del | (Flanigan et al., 2009) [91], (Kaspar et al., 2009) [24] |
| deletion exons 5-9 | c.265-?_960+?del | LBGM, Paris*, (Kaspar et al., 2009) [24] |
| deletion exons 5-13 | c.265-?_1602+?del | (Flanigan et al., 2009) [91] |
| deletion exons 5-37 | c.265-?_5325+?del | (Dent et al., 2005) [93] |
| deletion exons 5-44 | c.265-?_6438+?del | (Flanigan et al., 2009) [91] |
| deletion exons 5-48 | c.265-?_7098+?del | LBGM, Paris** |
| deletion exons 6-8 | c.358-?_831+?del | (Morandi et al., 1995) [22] |
| deletion exons 6-12 | c.358-?_1482+?del | LBGM, Paris* |
| deletion exons 6-13 | c.358-?_1602+?del | (Beggs et al., 1991) [18], (Kaspar et al., 2009) [24], LBGM, Paris* |
| deletion exons 7-11 | c.531-?_1331+?del | LBGM, Paris* |
| deletion exons 8-19 | c.650-?_2380+?del | (Taylor et al., 2007) [92], LBGM, Paris*, (Flanigan et al., 2009) [91] |
| deletion exons 8-21 | c.650-?_2803+?del | (Flanigan et al., 2009) [91], (Taylor et al., 2007) [92] |
| deletion exons 9-18 | c.832-?_2292+?del | (Taylor et al., 2007) [92] |
| deletion exons 10-12 | c.961-?_1482+?del | (Flanigan et al., 2009) [91] |
| deletion exons 10-13 | c.961-?_1602+?del | LBGM, Paris* |
| deletion exons 10-16 | c.961-?_1992+?del | LBGM, Paris* |
| deletion exons 10-18 | c.961-?_2292+?del | LBGM, Paris* |
| deletion exons 10-26 | c.961-?_3603+?del | LBGM, Paris* |
| deletion exons 10-28 | c.961-?_3921+?del | LBGM, Paris* |
| deletion exons 10-29 | c.961-?_4071+?del | LBGM, Paris* |
| deletion exons 10-34 | c.961-?_4845+?del | LBGM, Paris* |
| deletion exons 10-37 | c.961-?_5325+?del | (Morandi et al., 1995) [22] |
| deletion exons 10-38 | c.961-?_5448+?del | LBGM, Paris* |
| deletion exons 10-41 | c.961-?_5922+?del | LBGM, Paris* |
| deletion exons 10-42 | c.961-?_6117+?del | (Flanigan et al., 2009) [91], LBGM, Paris*, (Beggs et al., 1991) [18] |
| deletion exons 10-44 | c.961-?_6438+?del | (Beggs et al., 1991) [18], (Nevo et al., 2003) [95], (Flanigan et al., 2009) [91] |
| deletion exons 10-48 | c.961-?_7098+?del | (Flanigan et al., 2009) [91] |
| deletion exons 11-27 | c.1150-?_3786+?del | LBGM, Paris* |
| deletion exons 11-29 | c.1150-?_4071+?del | LBGM, Paris* |
| deletion exons 11-30 | c.1150-?_4233+?del | LBGM, Paris* |
| deletion exons 12-43 | c.1332-?_6290+?del | (Flanigan et al., 2009) [91] |
| deletion exons 13-30 | c.1483-?_4233+?del | LBGM, Paris* |
| deletion exons 13-34 | c.1483-?_4845+?del | LBGM, Paris*, (Morandi et al., 1995) [22] |
| deletion exons 13-40 | c.1483-?_5739+?del | (Nevo et al., 2003) [95] |
| deletion exons 13-41 | c.1483-?_5922+?del | (Beggs et al., 1991) [18], LBGM, Paris*, (Morandi et al., 1995) [22] |
| deletion exons 13-42 | c.1483-?_6117+?del | (Taylor et al., 2007) [92] |
| deletion exons 13-44 | c.1483-?_6438+?del | (Morandi et al., 1995) [22] |
| deletion exons 14-15 | c.1603-?_1812+?del | (Taylor et al., 2007) [92] |
| deletion exons 14-41 | c.1603-?_5922+?del | LBGM, Paris* |
| deletion exons 16-29 | c.1813-?_4071+?del | (Comi et al., 1994) [20], (Morandi et al., 1995) [22] |
| deletion exons 17-24 | c.1993-?_3276+?del | LBGM, Paris* |
| deletion exons 17-29 | c.1993-?_4071+?del | LBGM, Paris* |
| deletion exons 17-34 | c.1993-?_4845+?del | (Taylor et al., 2007) [92], LBGM, Paris* |
| deletion exons 19-20 | c.2293-?_2622+?del | (Taylor et al., 2007) [92], (Dent et al., 2005) [93] |
| deletion exons 20-50 | c.2381-?_7309+?del | (Flanigan et al., 2009) [91] |
| deletion exon 24 | c.3163-?_3276+?del | (Flanigan et al., 2009) [91] |
| deletion exons 24-27 | c.3163-?_3786+?del | (Flanigan et al., 2009) [91] |
| deletion exons 26-28 | c.3433-?_3921+?del | LBGM, Paris* |
| deletion exons 26-30 | c.3433-?_4233+?del | LBGM, Paris* |
| deletion exons 28-29 | c.3787-?_4071+?del | (Taylor et al., 2007) [92] |
| deletion exons 28-44 | c.3787-?_6438+?del | LBGM, Paris*, (Flanigan et al., 2009) [91] |
| deletion exons 28-49 | c.3787-?_7200+?del | (Carsana et al., 2005) [96] |
| deletion exon 30 | c.4072-?_4233+?del | (Taylor et al., 2007) [92] |
| deletion exons 30-42 | c.4072-?_6117+?del | (Flanigan et al., 2009) [91] |
| deletion exons 31-57 | c.4234-?_8547+?del | (Flanigan et al., 2009) [91] |
| deletion exons 32-42 | c.4345-?_6117+?del | (Morandi et al., 1995) [22] |
| deletion exons 32-44 | c.4345-?_6438+?del | (Morandi et al., 1995) [22] |
| deletion exon 34 | c.4675-?_4845+?del | (Taylor et al., 2007) [92] |
| deletion exons 34-51 | c.4675-?_7542+?del | (Taylor et al., 2007) [92] |
| deletion exons 35-42 | c.4846-?_6117+?del | LBGM, Paris* |
| deletion exons 35-44 | c.4846-?_6438+?del | (Beggs et al., 1991) [18] |
| deletion exons 41-44 | c.5740-?_6438+?del | (Comi et al., 1994) [20] |
| deletion exons 42-44 | c.5923-?_6438+?del | (Morandi et al., 1995) [22] |
| deletion exons 43-44 | c.6118-?_6438+?del | (Taylor et al., 2007) [92] |
| deletion exons 45-46 | c.6439-?_6762+?del | (Dent et al., 2005) [93], (Flanigan et al., 2009) [91], (Beggs et al., 1991) [18], (Morandi et al., 1995) [22], LBGM, Paris* |
| deletion exons 45-47 | c.6439-?_6912+?del | (Beggs et al., 1991) [18], (Tsukamoto et al., 1991) [97], (Comi et al., 1994) [20], (Kaspar et al., 2009) [24], (Morandi et al., 1995) [22], LBGM, Paris* , (Taylor et al., 2007) [92], (Dent et al., 2005) [93], (Flanigan et al., 2009) [91] |
| deletion exons 45-48 | c.6439-?_7098+?del | (Janssen et al., 2005) [98], (Kaspar et al., 2009) [24], (Morandi et al., 1995) [22], LBGM, Paris*, (Taylor et al., 2007) [92], (Dent et al., 2005) [93], (Flanigan et al., 2009) [91], (Beggs et al., 1991) [18], (Comi et al., 1994) [20] |
| deletion exons 45-49 | c.6439-?_7200+?del | (Flanigan et al., 2009) [91], (Beggs et al., 1991) [18], (Comi et al., 1994) [20], (Kaspar et al., 2009) [24], (Morandi et al., 1995) [22], LBGM, Paris*, (Taylor et al., 2007) [92], (Dent et al., 2005) [93] |
| deletion exons 45-51 | c.6439-?_7542+?del | (Kaspar et al., 2009) [24], (Morandi et al., 1995) [22], LBGM, Paris*, (Taylor et al., 2007) [92], (Flanigan et al., 2009) [91], (Buzin et al., 2005) [99] |
| deletion exons 45-53 | c.6439-?_7872+?del | (Taylor et al., 2007) [92], (Comi et al., 1994) [20], (Flanigan et al., 2009) [91], (Beggs et al., 1991) [18], (Kaspar et al., 2009) [24], LBGM, Paris* |
| deletion exons 45-55 | c.6439-?_8217+?del | (Kaspar et al., 2009) [24], LBGM, Paris*, (Taylor et al., 2007) [92], (Tsukamoto et al., 1991) [97] |
| deletion exons 45-57 | c.6439-?_8547+?del | LBGM, Paris*, (Flanigan et al., 2009) [91] |
| deletion exons 45-60 | c.6439-?_9084+?del | (Taylor et al., 2007) [92] |
| deletion exons 45-79 | c.6439-?_11055+?del | (Flanigan et al., 2009) [91] |
| deletion exons 46-54 | c.6615-?_8027+?del | (Taylor et al., 2007) [92] |
| deletion exon 47 | c.6763-?_6912+?del | (Kaspar et al., 2009) [24], (Taylor et al., 2007) [92] |
| deletion exons 47-48 | c.6763-?_7098+?del | (Kaspar et al., 2009) [24] |
| deletion exons 47-49 | c.6763-?_7200+?del | (Taylor et al., 2007) [92] |
| deletion exons 47-51 | c.6763-?_7542+?del | (Taylor et al., 2007) [92] |
| deletion exons 47-60 | c.6763-?_9084+?del | (Morandi et al., 1995) [22] |
| deletion exon 48 | c.6913-?_7098+?del | (Taylor et al., 2007) [92], (Ramelli et al., 2006) [100], (Kaspar et al., 2009) [24], (Beggs et al., 1991) [18], LBGM, Paris*, (Comi et al., 1994) [20], (Flanigan et al., 2009) [91] |
| deletion exons 48-49 | c.6913-?_7200+?del | LBGM, Paris*, (Comi et al., 1994) [20], (Morandi et al., 1995) [22], (Flanigan et al., 2009) [91], (Taylor et al., 2007) [92], (Kaspar et al., 2009) [24], (Beggs et al., 1991) [18] |
| deletion exons 48-51 | c.6913-?_7542+?del | (Morandi et al., 1995) [22], (Taylor et al., 2007) [92], (Kaspar et al., 2009) [24], (Beggs et al., 1991) [18], LBGM, Paris* |
| deletion exons 48-53 | c.6913-?_7872+?del | (Kaspar et al., 2009) [24], LBGM, Paris*, (Comi et al., 1994) [20] |
| deletion exons 48-55 | c.6913-?_8217+?del | LBGM, Paris* |
| deletion exons 48-59 | c.6913-?_8937+?del | LBGM, Paris* |
| deletion exon 49 | c.7099-?_7200+?del | LBGM, Paris* |
| deletion exons 49-51 | c.7099-?_7542+?del | LBGM, Paris*, (Kaspar et al., 2009) [24] |
| deletion exons 49-79 | c.7099-?_11055+?del | LBGM, Paris* |
| deletion exons 50-51 | c.7201-?_7542+?del | (Kaspar et al., 2009) [24], (Morandi et al., 1995) [22] |
| deletion exons 50-53 | c.7201-?_7872+?del | (Comi et al., 1994) [20], (Flanigan et al., 2009) [91] |
| deletion exons 50-79 | c.7201-?_11055+?del | LBGM, Paris* |
| deletion exons 51-52 | c.7310-?_7660+?del | LBGM, Paris*, (Taylor et al., 2007) [92], (Kaspar et al., 2009) [24] |
| deletion exons 51-76 | c.7310-?_10921+?del | LBGM, Paris* |
| deletion exons 52-59 | c.7543-?_8937+?del | (Flanigan et al., 2009) [91] |
| deletion exons 52-79 | c.7543-?_11055+?del | LBGM, Paris* |
| deletion exons 58-59 | c.8548-?_8937+?del | (Janssen et al., 2005) [98] |
| deletion exon 60 | c.8938-?_9084+?del | (Dent et al., 2005) [93], (Taylor et al., 2007) [92] |
| deletion exons 61-69 | c.9085-?_10086+?del | LBGM, Paris* |
| deletion exons 61-79 | c.9085-?_11055+?del | (Flanigan et al., 2009) [91] |
| deletion exon 64 | c.9287-?_9361+?del | (Flanigan et al., 2009) [91] |
| deletion exons 64-79 | c.9287-?_11055+?del | (Flanigan et al., 2009) [91] |
| deletion 482..484 | c.482-?_484+?del | LBGM, Paris* |
| deletion 676..678 | c.676_678del | (Flanigan et al., 2009) [91], LBGM, Paris* |
| deletion 2293..2379 | c.2293-?_2379+?del | LBGM, Paris* |
| deletion 2836..2838 | c.2836_2838del | (Flanigan et al., 2009) [91] |
| deletion 6647..7120 | c.6647_7120del | (Fajkusova et al., 2001) [101] |
| deletion 6647..8425 | c.6647_8425del | (Fajkusova et al., 2001) [101] |
| deletion 7121..7306 | c.7121_7306del | (Fajkusova et al., 2001) [101] |
| deletion 10097..10099 | c.10097_10099del | (Flanigan et al., 2009) [91] |
| duplication exons 2-7 | c.32-?_649+?dup | LBGM, Paris*, (Beggs et al., 1991) [18] |
| duplication exon 3 | c.94-?_186+?dup | (Flanigan et al., 2009) [91], (Taylor et al., 2007) [92], LBGM, Paris* |
| duplication exons 3-4 | c.94-?_264+?dup | (Taylor et al., 2007) [92], LBGM, Paris*, (Flanigan et al., 2009) [91] |
| duplication exons 3-5 | c.94-?_357+?dup | (Janssen et al., 2005) [98], LBGM, Paris* |
| duplication exons 3-8 | c.94-?_831+?dup | (Taylor et al., 2007) [92] |
| duplication exons 3-9 | c.94-?_960+?dup | LBGM, Paris* |
| duplication exons 3-13 | c.94-?_1602+?dup | (Dent et al., 2005) [93] |
| duplication exons 3-16 | c.94-?_1992+?dup | LBGM, Paris* |
| duplication exons 3-20 | c.94-?_2622+?dup | (Flanigan et al., 2009) [91] |
| duplication exons 3-26 | c.94-?_3603+?dup | LBGM, Paris* |
| duplication exons 3-29 | c.94-?_4071+?dup | (Flanigan et al., 2009) [91] |
| duplication exons 3-30 | c.94-?_4233+?dup | (Flanigan et al., 2009) [91] |
| duplication exons 3-34 | c.94-?_4845+?dup | (Flanigan et al., 2009) [91] |
| duplication exons 3-41 | c.94-?_5922+?dup | (Flanigan et al., 2009) [91] |
| duplication exons 3-44 | c.94-?_6438+?dup | LBGM, Paris* |
| duplication exon 4 | c.187-?_264+?dup | LBGM, Paris* |
| duplication exons 4-8 | c.187-?_831+?dup | LBGM, Paris* |
| duplication exon 5 | c.265-?_357+?dup | (Taylor et al., 2007) [92] |
| duplication exons 5-27 | c.265-?_3786+?dup | (Flanigan et al., 2009) [91] |
| duplication exons 8-19 | c.650-?_2380+?dup | (Taylor et al., 2007) [92] |
| duplication exons 9-41 | c.832-?_5922+?dup | (Janssen et al., 2005) [98] |
| duplication exons 10-37 | c.961-?_5325+?dup | LBGM, Paris* |
| duplication exons 13-16 | c.1483-?_1992+?dup | LBGM, Paris* |
| duplication exons 13-42 | c.1483-?_6117+?dup | (Beggs et al., 1991) [18] |
| duplication exons 14-18 | c.1603-?_2292+?dup | (Beggs et al., 1991) [18], LBGM, Paris* |
| duplication exons 14-29 | c.1603-?_4071+?dup | LBGM, Paris* |
| duplication exons 14-53 | c.1603-?_7872+?dup | LBGM, Paris* |
| duplication exons 16-27 | c.1813-?_3786+?dup | LBGM, Paris* |
| duplication exons 16-42 | c.1813-?_6117+?dup | (Flanigan et al., 2009) [91] |
| duplication exons 17-44 | c.1993-?_6438+?dup | LBGM, Paris*, (Taylor et al., 2007) [92] |
| duplication exons 19-30 | c.2293-?_4233+?dup | LBGM, Paris* |
| duplication exons 19-44 | c.2293-?_6438+?dup | (Flanigan et al., 2009) [91] |
| duplication exons 21-55 | c.2623-?_8217+?dup | LBGM, Paris* |
| duplication exon 27 | c.3604-?_3786+?dup | (Flanigan et al., 2009) [91] |
| duplication exons 43-47 | c.6118-?_6912+?dup | LBGM, Paris* |
| duplication exons 43-51 | c.6118-?_7542+?dup | (Taylor et al., 2007) [92] |
| duplication exons 45-48 | c.6439-?_7098+?dup | (Taylor et al., 2007) [92] |
| duplication exons 45-48+dup 54-55 | c.[6439-?_7098+?dup(+)7873-?_8217+?dup] | (Janssen et al., 2005) [98] |
| duplication exons 45-49 | c.6439-?_7200+?dup | (Taylor et al., 2007) [92], LBGM, Paris* |
| duplication exons 45-51 | c.6439-?_7542+?dup | (Buzin et al., 2005) [99] |
| duplication exons 45-55 | c.6439-?_8217+?dup | LBGM, Paris* |
| duplication exons 48-49 | c.6913-?_7200+?dup | LBGM, Paris* |
| duplication exons 50-51 | c.7201-?_7542+?dup | (Taylor et al., 2007) [92] |
| duplication exons 50-55 | c.7201-?_8217+?dup | (Flanigan et al., 2009) [91], LBGM, Paris* |
| duplication exons 50-60 | c.7201-?_9084+?dup | (Taylor et al., 2007) [92], LBGM, Paris* |
| duplication exons 51-52 | c.7310-?_7660+?dup | (Taylor et al., 2007) [92] |
| duplication exons 52-59 | c.7543-?_8937+?dup | LBGM, Paris* |
| duplication exons 54-55 | c.7873-?_8217+?dup | LBGM, Paris* |
| duplication exons 54-57 | c.7873-?_8547+?dup | (Flanigan et al., 2009) [91] |
| duplication exons 56-60 | c.8218-?_9084+?dup | LBGM, Paris* |
| duplication exon 60 | c.8938-?_9084+?dup | (Janssen et al., 2005) [98] |
| substitution 137A>T | c.137A>T | LBGM, Paris* |
| substitution 161T>G | c.161T>G | (Prior and Bridgeman, 2005) [102] |
| substitution 347T>C | c.347T>C | (Flanigan et al., 2009) [91] |
| substitution 474C>G | c.474C>G | (Taylor et al., 2007) [92] |
| substitution 494A>T | c.494A>T | (Flanigan et al., 2009) [91] |
| substitution 596C>A | c.596C>A | (Flanigan et al., 2009) [91] |
| substitution 653T>G | c.653T>G | LBGM, Paris* |
| substitution 691T>G | c.691T>G | (Flanigan et al., 2009) [91] |
| substitution 1351G>T | c.1351G>T | LBGM, Paris* |
| substitution 1934A>G | c.1934A>G | (Prior and Bridgeman, 2005) [102] |
| substitution 2378A>G | c.2378A>G | LBGM, Paris* |
| substitution 2795T>C | c.2795T>C | (Ramelli et al., 2006) [100] |
| substitution 3952G>C | c.3952G>C | (Taylor et al., 2007) [92] |
| substitution 8219A>G | c.8219A>G | (Flanigan et al., 2009) [91] |
| substitution 8390G>A | c.8390G>A | LBGM, Paris* |
| substitution 8668G>A | c.8668G>A | LBGM, Paris* |
| substitution 9560A>G | c.9560A>G | (Taylor et al., 2007) [92] |
| substitution 9619T>C | c.9619T>C | (Taylor et al., 2007) [92] |
| substitution 9938G>T | c.9938G>T | (Flanigan et al., 2009) [91] |
| substitution 9955T>C | c.9955T>C | LBGM, Paris* |
| substitution 10003G>A | c.10003G>A | (Taylor et al., 2007) [92] |
| substitution 10102G>C | c.10102G>C | LBGM, Paris* |
| substitution 10103A>G | c.10103A>G | (Flanigan et al., 2009) [91] |

*LBGM, Paris : Laboratoire de Biochimie et Génétique Moléculaire–Hôpital Cochin, Paris, France.
