# Supplementary figures and images for: Assessment of the structural and functional impact of in-frame mutations of the DMD gene, using the tools included in the eDystrophin online database
Source: Orphanet J Rare Dis. 2012 Jul 9;7:45. doi: 10.1186/1750-1172-7-45 (PMC3748829; doi:10.1186/1750-1172-7-45)

Additional figure S1

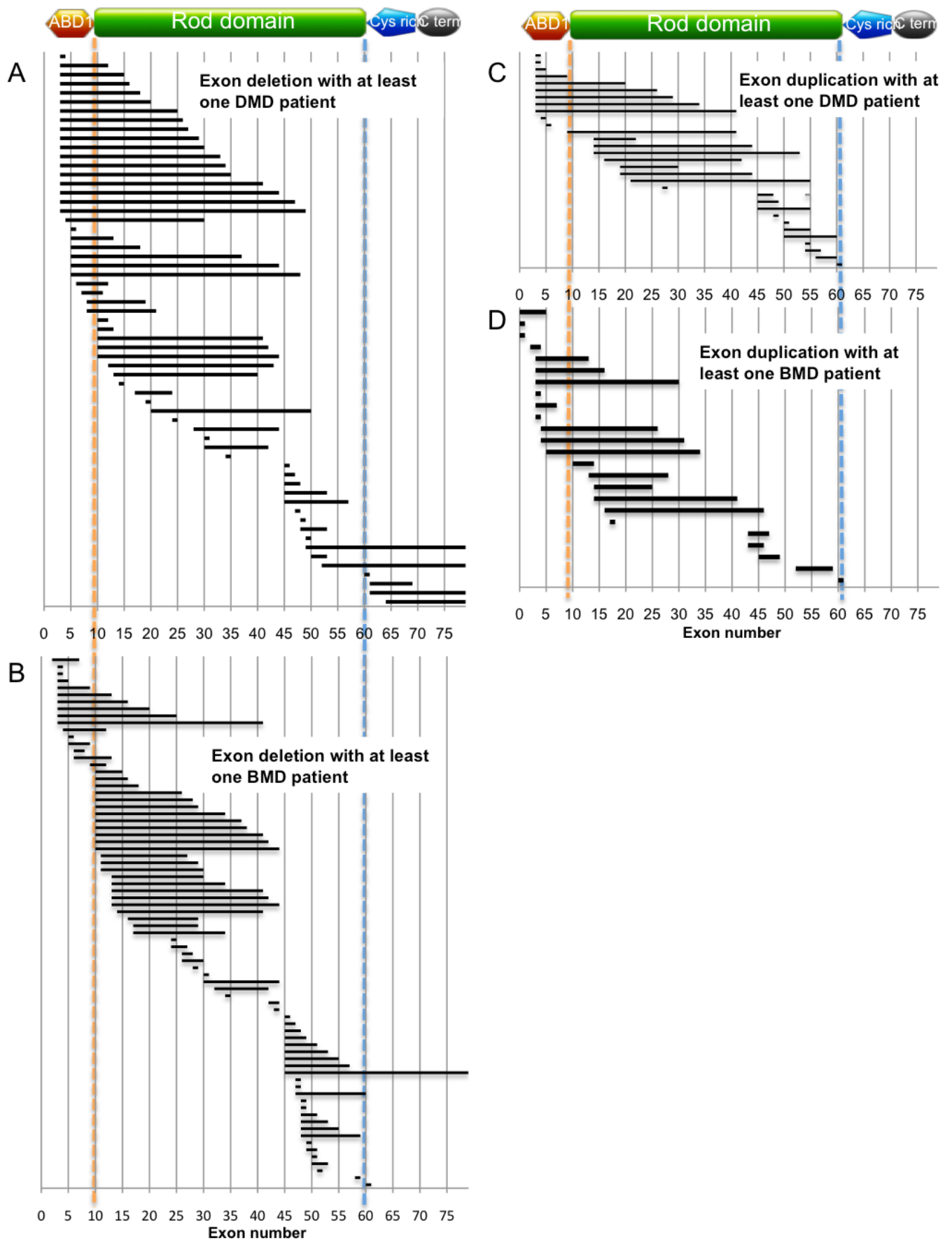

Supplement: Additional file 3 Figure S1. — Statistics for mutations included in eDystrophin. Exon deletions (A, B) and duplications (C, D) associated with at least one DMD (A, C) or one BMD (B, D) patient. Each line represents a type of exon deletion (A and B) or duplication (C and D). [file 1750-1172-7-45-S3.pdf]
