# Supplementary material for: Assessment of the structural and functional impact of in-frame mutations of the DMD gene, using the tools included in the eDystrophin online database
Source: Orphanet J Rare Dis. 2012 Jul 9;7:45. doi: 10.1186/1750-1172-7-45 (PMC3748829; doi:10.1186/1750-1172-7-45)
Supplement: Additional file 4 Figure S2. — Sequence alignment of the 24 spectrin-like repeats of dystrophin. Repeats were aligned by ClustalW, using default parameters, as described by Winder et al.[7]. The alignment was visualized in Jalview. In the first line, heptad motifs are indicated, showing the hydrophobic residues in the (a) and (d) positions. The repeat numbers and the number of residues per repeat are indicated at the start of the repeat sequence. The presence of absence of hinges 2 and 3 is indicated in separate lines. There are two extra sequences at the ends of repeats 15 and 18 not aligned with the heptad pattern. The presence of these sequences is indicated at the end of the corresponding lines. At the end of each line, the numbers of the exons encoding the repeat are indicated in parentheses. The repeat sequences are highlighted alternately in orange (even-numbered exons) and light yellow (odd-numbered exons). The rectangle indicates the middle of the B helices of the repeats. If the successive exons are not in frame, the residues are shown in red. The alignment reported by Koenig et al.[76] is also mentioned: the residues at the start of the repeats are underlined when they differ from Winder’s alignment. [file 1750-1172-7-45-S4.pdf]

# Additional figure S2

```
                        Helix A                                     Helix B                                        Helix C
                  <------------------------->              <------------------------------------------>          <------------------------------>
              ...a..d...a..d...a..d...a...          a..d...a..d...abcdefga..d...a..d...a..d.....          a..d...a..d...a..d...a..d...a
R1/1-109      sEVNLDRYQTALEEVLSWLLSAEDTLQA------QGEISNDVEVVKDQFHTHEGYMMDLTAHQGRVGNILQLGSKLIGTGK--LSEDEETEVQEQMNLLNSRWECLRVASMEKQSNLH----(10, 11, 12)
R2/1-111       RVLMDLQNQKLKELNDWLTKTEERTRK----MEEEPLGPDLEDLKRQVQQHKVLQEDLEQEQVRVNSLTHMVVVVDESSG----DHATAALEEQLKVLGDRWANICRWTEDRWVLLQDI– (12, 13, 14)
R3/1-111       LLKWQRLTEEQCLFSAWLSEKEDAVNK-----IHTTGFKDQNEMLSSLQKLAVLKADLEKKKQSMGKLYSLKQDLLSTLK---NKSVTQKTEAWLDNFARCWDNLVQKLEKSTAQISQA– (14, 15, 16, 17)
              <HINGE 2>                                                                                                                     (17)
R4/1-111       SEIRKRLDVDITELHSWITRSEAVLQS-----PEFAIFRKEGNFSDLKEKVNAIEREKAEKFRKLQDASRSAQALVEQMV--NEGVNADSIKQASEQLNSRWIEFCQLLSERLNWLEY--- (17, 18, 19, 20)
R5/1-109       QNNIIAFYNQLQQLEQMTTTAENWLKI------QPTTPSEPTAIKSQLKICKDEVNRLSGLQPQIERLKIQSIALKEKGQ------GPMFLDADFVAFTNHFKQVFSDVQAREKELQTIFD (20, 21, 22)
R6/1-109       TLPPMRYQETMSAIRTWVQQSETKLSI------PQLSVTDYEIMEQRLGELQALQSSLQEQQSGLYYLSTTVKEMSKKAP----SEISRKYQSEFEEIEGRWKKLSSQLVEHCQKLEEQ–  (22, 23)
R7/1-109       MNKLRKIQNHIQTLKKWMAEVDVFLKE------EWPALGDSEILKKQLKQCRLLVSDIQTIQPSLNSVNEGGQKIKNEAE----PEFASRLETELKELNTQWDHMCQQVYARKEALKGG–  (23, 24, 25, 26)
R8/1-109       LEKTVSLQKDLSEMHEWMTQAEEEYLE------RDFEYKTPDELQKAVEEMKRAKEEAQQKEAKVKLLTESVNSVIAQAP----PVAQEALKKELETLTTNYQWLCTRLNGKCKTLEEV–  (26, 27, 28)
R9/1-104       WACWHELLSYLEKANKWLNEVEFKLKT---------TENIPGGAEEISEVLDSLENLMRHSEDNPNQIRILAQTLTDGGV------MDELINEELETFNSRWRELHEEAVRRQKLLEQS–  (28, 29, 30)
R10/1-92       IQSAQETEKSLHLIQESLTFIDKQLAA---------YIADKVDAAQMPQEAQKIQSDLTSHEISLEEMKKHNQGKE-----------------AAQRVLSQIDVAQKKLQDVSMKFRL---  (30, 31, 32)
R11/1-109      FQKPANFEQRLQESKMILDEVKMHLPA------LETKSVEQEVVQSQLNHCVNLYKSLSEVKSEVEMVIKTGRQIVQKKQ---TENPKELDERVTALKLHYNELGAKVTERKQQLEKC–  (32, 33, 34)
R12/1-108      LKLSRKMRKEMNVLTEWLAATDMELTK------RSAVEGMPSNLDSEVAWGKATQKEIEKQKVHLKSITEVGEALKTVLG-----KKETLVEDKLSLLNSNWIAVTSRAEEWLNLLLEY–  (34, 35, 36)
R13/1-104      QKHMETFDQNVDHITKWIIQADTLLDE-----------SEKKKPQQKEDVLKRLKAELNDIRPKVDSTRDQAANLMANRG----DHCRKLVEPQISELNHRFAAISHRIKTGKASIPLK–  (36, 37, 38)
R14/1-94       --ELEQFNSDIQK---LLEPLEAEIQQ-------GVNLKEEDFNKDMNEDNEGTVKELLQRGDNLQQRITDERKREE-------------IKIKQQLLQTKHNALKDLRSQRRKKALEI–  (38, 39, 40)
R15/1-98       SHQWYQYKRQADDLLKCLDDIEKKLAS--------------LPEPRDERKIKEIDRELQKKKEELNAVRRQAEGLSEDG--------AAMAVEPTQIQLSKRWREIESKFAQFRRLNFAQ-<18> (40, 41, 42)
R16/1-109     EISYVPSTYLTEITHVSQALLEVEQLLNA------PDLCAKDFEDLFKQEESLKNIKDSLQQSSGRIDIIHSKKTAALQSAT----PVERVKLQEALSQLDFQWEKVNKMYKDRQGRFDRS–  (42, 43, 44)
R17/1-107      VEKWRRFHYDIKIFNQWLTEAEQFLRK--------TQIPENWEHAKYKWYLKELQDGIGQRQTVVRTLNATGEEIIQQSS----KTDASILQEKLGSLNLRWQEVCKQLSDRKKRLEEQ–  (44, 45, 46)
R18/1-110      KNILSEFQRDLNEFVLWLEEADNIASI-------PLEPGKEQQLKEKLEQVKLLVEELPLRQGILKQLNETGGPVLVSAP--ISPEEQDKLENKLKQTNLQWIKVSRALPEKQGEIEAQ–<7>(46, 47, 48)
R19/1-94      QLEKKLEDLEEQLNHLLLWLSPIRNQLEI------------YNQPNQEGPFDVQETEIAVQAKQPDVEEILSKGQHLYKEKP------ATQPVKRKLEDLSSEWKAVNRLLQELRAKQPDL-  (48, 49, 50)
              <HINGE 3>                                                                                                                     (50, 51)
R20/1-110      VPALADFNRAWTELTDWLSLLDQVIKS------QRVMVGDLEDINEMIIKQKATMQDLEQRRPQLEELITAAQNLKNKTS---NQEARTIITDRIERIQNQWDEVQEHLQNRRQQLNEM–   (51, 52, 53)
R21/1-109      LKDSTQWLEAKEEAEQVLGQARAKLES------WKEGPYTVDAIQKKITETKQLAKDLRQWQTNVDVANDLALKLLRDYS----ADDTRKVHMITENINASWRSIHKRVSEREAALEET–   (53, 54, 55)
R22/1-112      HRLLQQFPLDLEKFLAWLTEAETTANVLQDATRKERLLEDSKGVKELMKQWQDLQGEIEAHTDVYHNLDENSQKILRSLE---GSDDAVLLQRRLDNMNFKWSELRKKSLNIRSHLEAS–   (55, 56, 57)
R23/1-109      SDQWKRLHLSLQELLVWLQLKDDELSR------QAPIGGDFPAVQKQNDVHRAFKRELKTKEPVIMSTLETVRIFLTEQP<20>QNVTRLLRKQAEEVNTEWEKLNLHSADWQRKIDET---  (57, 58, 59)
R24/1-108      LERLQELQEATDELDLKLRQAEVIKGS-----WQPVGDLLIDSLQDHLEKVKALRGEIAPLKENVSHVNDLARQLTTLGI-----QLSPYNLSTLEDLNTRWKLLQVAVEDRVRQLHE----  (59, 60, 61)
```
